# Role of Endolysosomes in Severe Acute Respiratory Syndrome Coronavirus-2 Infection and Coronavirus Disease 2019 Pathogenesis: Implications for Potential Treatments

**DOI:** 10.3389/fphar.2020.595888

**Published:** 2020-10-29

**Authors:** Nabab Khan, Xuesong Chen, Jonathan D. Geiger

**Affiliations:** Department of Biomedical Sciences, University of North Dakota School of Medicine and Health Sciences, Grand Forks, ND, United States

**Keywords:** endolysosome, endocytosis, two pore channel

## Abstract

Severe acute respiratory syndrome coronavirus-2 (SARS-CoV-2) is an enveloped, single-stranded RNA virus. Humans infected with SARS-CoV-2 develop a disease known as coronavirus disease 2019 (COVID-19) with symptoms and consequences including acute respiratory distress syndrome (ARDS), cardiovascular disorders, and death. SARS-CoV-2 appears to infect cells by first binding viral spike proteins with host protein angiotensin-converting enzyme 2 (ACE2) receptors; the virus is endocytosed following priming by transmembrane protease serine 2 (TMPRSS2). The process of virus entry into endosomes and its release from endolysosomes are key features of enveloped viruses. Thus, it is important to focus attention on the role of endolysosomes in SARS-CoV-2 infection. Indeed, coronaviruses are now known to hijack endocytic machinery to enter cells such that they can deliver their genome at replication sites without initiating host detection and immunological responses. Hence, endolysosomes might be good targets for developing therapeutic strategies against coronaviruses. Here, we focus attention on the involvement of endolysosomes in SARS-CoV-2 infection and COVID-19 pathogenesis. Further, we explore endolysosome-based therapeutic strategies to restrict SARS-CoV-2 infection and COVID-19 pathogenesis.

## Introduction

Severe acute respiratory syndrome-coronavirus-2 (SARS-CoV-2) that causes the pandemic disease known as coronavirus disease 2019 (COVID-19) (Contini et al., 2020; Gudbjartsson et al., 2020) is an enveloped virus that contains a large single-stranded RNA genome (Chan et al., 2020; Huang et al., 2020; Lu et al., 2020; Ren et al., 2020). SARS-CoV-2 belongs to the same beta-coronavirus family as does SARS-CoV that caused the SARS outbreak in China in 2002 (Cherry, 2004) and Middle East respiratory syndrome coronavirus (MERS-CoV) that caused the MERS outbreak in Saudi Arabia in 2012 (Zaki et al., 2012; Li and Du, 2019). Similar to other enveloped coronaviruses, SARS-CoV-2 enters host cells by endocytosis and uses host cell machinery for replication.

Spiked glycoproteins on the outer surface of coronaviruses are recognized by and bind to cell surface receptors such as angiotensin-converting enzyme 2 (ACE2) (Huang et al., 2006; Hoffmann et al., 2020b; Shang et al., 2020b) as well as possibly other co-receptors (Raj et al., 2013). Following binding, receptor-bound virus is endocytosed whereupon the viral genome is delivered into the cytoplasm; endocytosis mechanisms are pH-dependent and -independent (Dimitrov, 2004; White and Whittaker, 2016). Viruses that co-opt pH-independent mechanisms, an example of which is HIV-1, fuse with cell surface membranes and use endocytic pathways to achieve infection (White and Whittaker, 2016). Viruses that enter cells by pH-dependent mechanisms fuse with endosome membranes and use host factors associated with endosomes to enable viral entry into cells (Yang et al., 2004; White and Whittaker, 2016).

Coronaviruses use endolysosome-associated cathepsin B and L proteases under acidic conditions and are considered to be late penetrating viruses (late-entry kinetic mechanism) (Follis et al., 2006; Bosch et al., 2008; Millet and Whittaker, 2014; Coutard et al., 2020; Hoffmann et al., 2020a; Hoffmann et al., 2020b; Pranesh et al., 2020). Following entry, coronaviruses are released into the cytosol from endolysosomes or are targeted for degradation in lysosomes. In addition, some coronaviruses including SARS-CoV-2 can escape endolysosomes and replicate in autophagosome-like structures in the cytosol (Maier and Britton, 2012; Chen et al., 2014; Gassen et al., 2019; Gassen et al., 2020). Accordingly, it is important to focus attention on the role of endolysosomes in early stages of interactions between the virus and host cells as well as COVID-19 pathogenesis.

## The Acidic Nature of Endolysosomes

**FIGURE 1:**
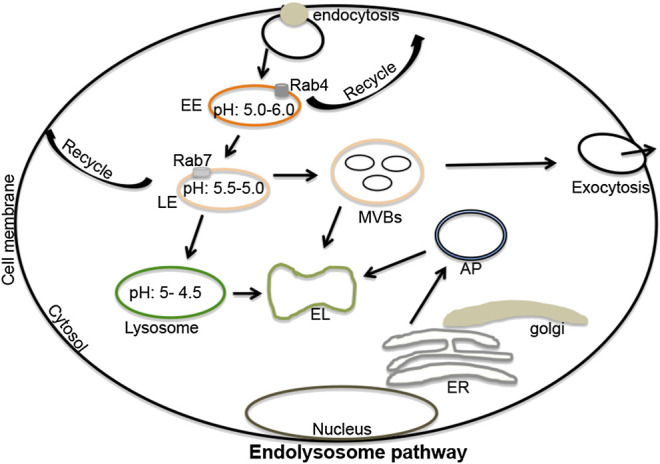
The endolysosome pathway: Extracellular signaling molecules upon binding to cell surface receptors can be engulfed by endocytosis. These endocytosed vesicles can mature and differentiate into early endosomes (pH 5.5–6.0), late endosomes (pH 5.5–5.0), lysosomes (pH 5.0–4.5), and endolysosomes (a fusion process of lysosomes and late endosomes). Various marker substances can differentiate early from late endosomes including Rab4 (early endosomes), and Rab5 and Rab7 (late endosomes). Both early and late endosomes regulate recycling processes that return constituent molecules back to plasma membranes. Late endosomes can produce multi-vesicular bodies, which can fuse with lysosomes or can be released from cells in the form of extracellular vesicles (exosomes). Lysosomes regulate the degradation of extracellular materials in endolysosomes produced by fusions with late endosomes. Lysosomes can also fuse with autophagosomes to form autolysosomes; sites where extracellular and intracellular components are degraded. EL, endolysosomes; ER, endoplasmic reticulum; EE, early endosomes; LE, late-endosomes; MVBs, multi-vesicular bodies; AP, autophagosomes; Rab, ras-related protein 4, 5 and 7).

Endosomes are formed from plasma membrane invaginations; a process known as endocytosis. These acidic organelles are categorized further as early, late and recycling endosomes; all with different compositions and hydrogen ion (H^+^) content (Luzio et al., 2007; Huotari and Helenius, 2011; Gautreau et al., 2014). Rab4 and Rab5 are important components of early endosomes and function optimally at a pH range of 5.5–6.0. Early endosomes participate in signaling between the extracellular and intracellular environments (Pálfy et al., 2012; Villaseñor et al., 2016); they can recycle to plasma membranes thereby returning endocytosed constituents back to the cell surface (McCaffrey et al., 2001; Grant and Donaldson, 2009; Hsu and Prekeris, 2010). Alternatively, early endosomes can mature and transform into late endosomes (Bright et al., 2005; Luzio et al., 2007); these are differentiated from early endosomes by the expression of Rab7 and have an optimal pH range of 5.0–5.5 (Vanlandingham and Ceresa, 2009; Guerra and Bucci, 2016). Late endosomes can also recycle to plasma membranes (Guerra and Bucci, 2016), can produce multi-vesicular bodies from which extracellular vesicles (exosomes) originate, or can fuse with lysosomes (Piper and Luzio, 2001; Traub, 2010). The fusion of late endosomes with lysosomes generates endolysosomes under more acidic conditions ranging from pH 4.5–5.0 (Figure 1) (Mullock et al., 1998; Luzio et al., 2007; Luzio et al., 2010). The tight range of H^+^ concentrations in these organelles controls enzymatic activities as well as fusions between autophagolysosomes and lysosomes, and lysosomes and endosomes; pH also affects autophagy and other important cellular processes (Luzio et al., 2007; Luzio et al., 2010; Nakamura and Yoshimori, 2017). Vacuolar-ATPase (v-ATPase) activity largely regulates the acidic nature of endolysosomes and does so by controlling the flux of cations and anions via hydrolysis of free ATP that drives protons against their electrochemical gradient into the lumen of endolysosomes (Mindell, 2012; Halcrow et al., 2019a; Khan et al., 2019a).

Endolysosomes are involved in a wide range of cellular processes including membrane trafficking, catabolism of extracellular and intracellular components, immune responses and antigen presentation, cell secretions, and cell life and death (Eskelinen and Saftig, 2009; Munz, 2012; Repnik et al., 2013; Bright et al., 2016; Truschel et al., 2018; Khan et al., 2019a; Afghah et al., 2020). These acidic organelles have also been implicated in various pathological conditions; structural and functional changes have been reported in various neurodegenerative disorders as well as in cancer (Repnik et al., 2013; Bright et al., 2016; Davis, 2018; Halcrow et al., 2019a; Khan et al., 2019a). Because endolysosome pH regulates structural and functional features of endolysosomes, the involvement of v-ATPase in disease pathogenesis has received much attention and the v-ATPase complex has been targeted for therapeutic reasons. Indeed, inhibitors of v-ATPase and other strategies to keep endolysosomes from de-acidifying has shown benefit against diverse pathological conditions including different types of cancer (Whitton et al., 2018; Halcrow et al., 2019a; Halcrow et al., 2019b), neurological complications (Colacurcio and Nixon, 2016), and infectious diseases (Luzio et al., 2007).

## Coronavirus Entry Into and Escape From Endolysosomes:

Coronaviruses once endocytosed can avoid immune surveilence detection and degradation; thus enhancing infection (Hofmann and Pöhlmann, 2004; Belouzard et al., 2012; Shang et al., 2020a; Letko et al., 2020; Stower, 2020). SARS-CoV and MERS-CoV bind principally to dipeptidyl peptidase 4 while SARS-CoV-2 appears to bind mainly to ACE2; regardless, coronavirus spike proteins are activated by the host proteases TMPRSS2 or cathepsin B/L (Bosch et al., 2008; Shirato et al., 2013; Zhou et al., 2015; Hoffmann et al., 2020b; Pranesh et al., 2020). In addition, SARS-CoV-2 and MERS-CoV are activated by furin and this enhances viral entry especially in cells with lower expression levels of lysosomal cathepsin (Follis et al., 2006; Millet and Whittaker, 2014; Coutard et al., 2020; Hoffmann et al., 2020a).

Coronaviruses enter host cells by pH-dependent endocytosis (Yang et al., 2004; Burkard et al., 2014; Hoffmann et al., 2020b) and the acidic environment of endolysosomes is regulated not only by v-ATPase (Mindell, 2012), but also by Na^+^/K^+^-ATPase (Cain et al., 1989), mucolipin (TRPML1) channels (Li M. et al., 2017), big potassium channels (BK and MaxiK) (Khan et al., 2019b), Niemann-Pick type C (NPC1) (Wheeler et al., 2019a; Wheeler et al., 2019b; Höglinger et al., 2019; Lim et al., 2019), and two-pore channels (TPCs) (Marchant and Patel, 2015; Grimm et al., 2017; Khan et al., 2020). To date, TPCs and NPC1 have both been implicated in coronavirus infectivity.

**FIGURE 2:**
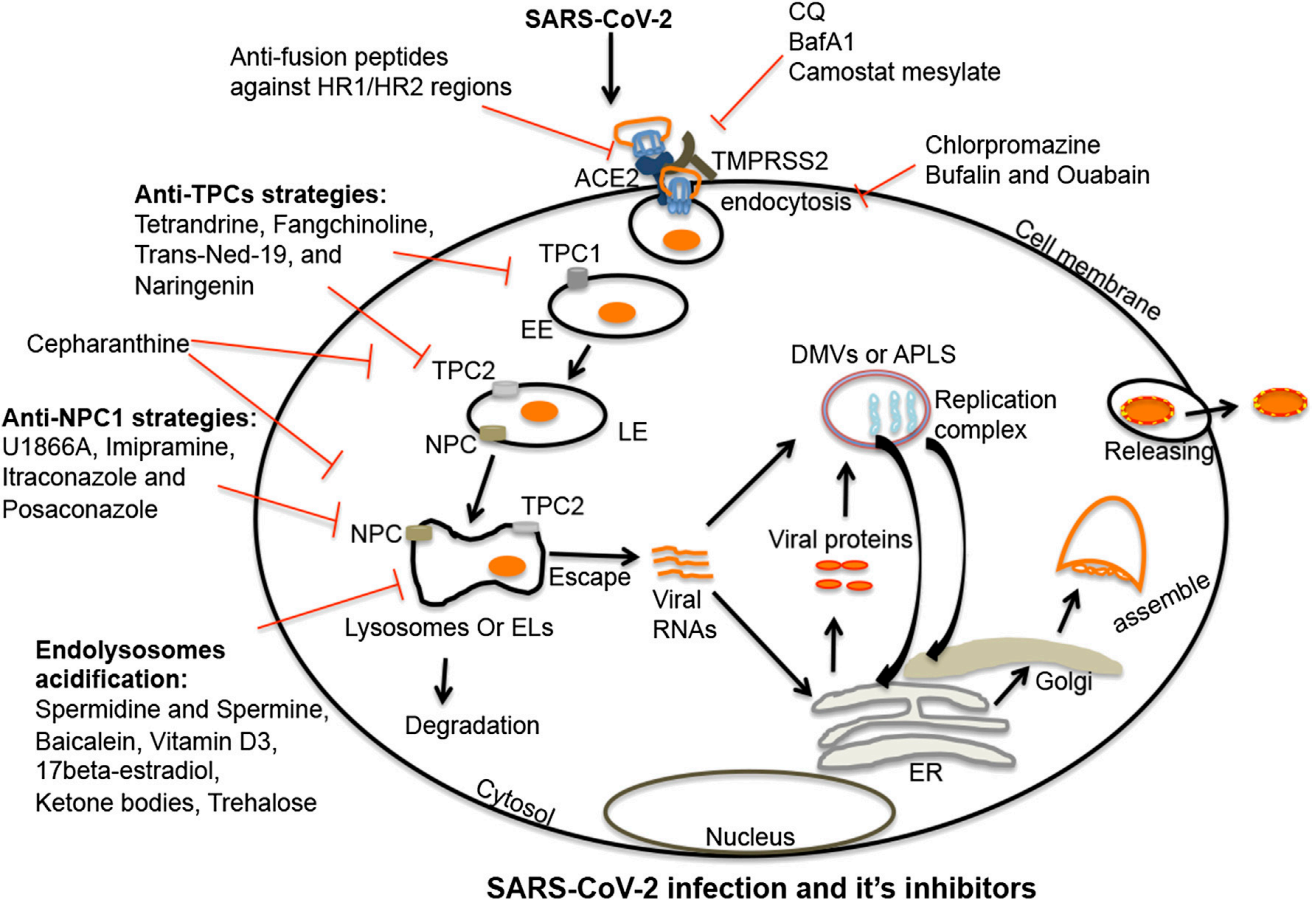
Endolysosome-mediated therapeutic strategies against SARS-CoV-2: SARS-CoV-2 enters cells following interactions between viral spike proteins and cell surface ACE2 receptors. Once endocytosed, spike proteins in endosomes are primed in late endosomes/lysosomes by cathepsin enzymes (B/L); this enhances virus entry. Post-fusion, virus is either released from or degraded in endolysosomes. SARS-CoV-2 once released from endolysosomes, enters the cytosol where it produces a replication complex to generate viral genomic and sub-genomic RNA. Following replication, viral structural proteins get inserted into the ER and move to the ERGIS (endoplasmic reticulum–Golgi intermediate compartment) secretory pathway for virus assembly. Following assembly, virions are transported to vesicles and released from cells by exocytosis. Thus, various stages are targetable for intervention. The first target might be fusion between spike proteins and host ACE2 receptors. A second target might be de-acidification of endolysosomes and blocking the priming of spike proteins by deactivating serine proteases. A third target might be clathrin-mediated endocytosis. Fourth, TPC and NPC1 inhibitors could effectively inhibit the virus infection by de-acidifying endolysosomes and blocking the trafficking of cholesterol. Endolysosome acidification may also be a therapeutic target because of its capacity to block the escape of viral RNA to the cytosol and enhance the degradation of the virus in lysosomes. Shown in the figure are multiple compounds and drugs capable of targeting each of these important steps in the virus cycle. SARS-CoV-2, severe acute respiratory syndrome coronavirus-2; TMPRSS2, transmembrane protease serine 2; ACE2, angiotensin-converting enzyme 2; EL, endolysosome; EE, early endosome; LE, late-endosome; TPC1/2, two-pore channel 1 and 2; NPC1, Niemann-Pick disease type C1; viral RNAs, viral ribonucleic acids; DMVs, double-membrane vesicles; APLS, autophagosome-like structures; ER, endoplasmic reticulum.

TPCs are present in two forms; TPC1 and TPC2. TPC1s are mainly localized on early endosomes while TPC2s are mainly found on late endosomes/lysosomes (Brailoiu et al., 2009; Pitt et al., 2010; Zakon, 2012). Both subtypes of TPCs can help orchestrate interactions between endolysosomes and such viruses as Ebola (Sakurai et al., 2015), MERS-CoV (Gunaratne et al., 2018), and SARS-CoV-2 (Ou et al., 2020); TPCs regulate the trafficking of virus to late-endosomes/lysosomes following entry into cells. Not surprisingly then, TPC inhibitors can block entry of SARS-CoV-2 into cells and restrict the release of viral RNA into the cytosol (Figure 2) (Ou et al., 2020). TPCs are also involved in chloroquine-mediated endolysosome leakage and facilitated the release of HIV-1 Tat protein from endolysosomes thus enabling activation of HIV-1 LTR transactivation in the nucleus (Khan et al., 2020). Therefore, TPCs appear to promote virus entry and facilitate the release and transport of viral RNA to replication sites by inducing endolysosome permeability and depolarization.

NPC1 appears to also play a role in virus entry and infectivity. SARS-CoV enters into early endosomes, traffics to NPC1-positive late endosomes and lysosomes, and accesses highly active cathepsin L protease that triggers fusion mechanisms (Figure 2) (Shah et al., 2010; Zheng et al., 2018). MERS-CoV, Ebola, and SARS-CoV-2 use similar mechanisms to enter into host cells (Mingo et al., 2015; Zhou et al., 2016; Ballout et al., 2020).

## Autophagy and Coronavirus Replication

Autophagy is a process by which extracellular and intracellular macromolecules are engulfed in and degraded by autophagolysosomes; structures formed by fusion of lysosomes with autophagosomes (Eskelinen and Saftig, 2009; Kenney and Benarroch, 2015; Yim and Mizushima, 2020). Autophagy is regulated by diverse proteins including autophagy-related-genes (ATGs), *Beclin*, ubiquitin-binding protein (p62), 5′-adenosine monophosphate-activated protein kinase, serine/threonine kinase 1 (Akt), and S-phase kinase-associated protein 2 (Skp2) (He and Klionsky, 2009; Badadani, 2012).

The process of autophagy degrades invading viruses, enhances antigen processing and presentation, and induces adaptive immune responses (Lee and Kim, 2007; Delgado et al., 2009; Richetta and Faure, 2013; Choi et al., 2018a). For example, toll-like receptors are pattern recognition receptors that sense viral RNA and DNA in endolysosomes, induce type I-interferon responses, and following induction of autophagy antiviral immune responses are decreased and invading viruses are degraded (Lee and Kim, 2007; Dalpke and Helm, 2012; Choi et al., 2018a). Autophagy has antiviral effects independent of the degradation process; interferon-γ can suppress replication of norovirus (Hwang et al., 2012; Baldridge et al., 2016; Biering et al., 2017). Additionally, viruses can modulate, escape, and inhibit autophagy at multiple steps to survive and replicate in host cells (Pattingre et al., 2005; Kyei et al., 2009; Chaumorcel et al., 2012).

Autophagy plays a role in viral infections including those caused by coronaviruses (Prentice et al., 2004a; Killian, 2012; Maier and Britton, 2012). Mouse hepatitis virus (MHV) has been used as a model for coronavirus infections (Prentice et al., 2004a); the replication complex of MHV generates double-membrane vesicles (DMVs) resembling autophagosomes (Snijder et al., 2006; Clementz et al., 2008; Gadlage et al., 2010) within which the autophagy markers LC3 and ATG12 colocalize (Prentice et al., 2004a). MHV replication is impaired when the autophagy marker ATG5 is knocked down (Prentice et al., 2004a). Replication proteins of SARS-CoV colocalize with LC3 and autophagy appears to play an important role in SARS-CoV replication (Prentice et al., 2004b). In contrast, SARS-CoV and MHV replication was not impaired when ATG5 and ATG7 were knocked down (Zhao et al., 2007; Reggiori et al., 2010; Schneider et al., 2012). The MERS-CoV and SARS-CoV associated protein, membrane-associated papain-like proteases, suppressed autophagy flux by blocking the fusion of lysosomes and autophagosomes (Chen X. et al., 2014; Gassen et al., 2019). Similarly, SARS-CoV-2 suppresses autophagy by modulating multiple autophagy regulatory factors (Gassen et al., 2020), by blocking the degradation of viral factors, and by increasing the formation of DMVs to promote virus replication. Induction of autophagy reduced the replication and infectivity of MERS-CoV (Gassen et al., 2019; Carmona-Gutierrez et al., 2020) and SARS-CoV-2 (Maier and Britton, 2012; Gassen et al., 2020).

## Endolysosome-Based Therapeutic Strategies to Inhibit Severe Acute Respiratory Syndrome Coronavirus-2 Infection

Because endolysosomes influence coronavirus infections, these organelles might be targeted against SARS-CoV-2 infection and COVID-19 pathogenesis. Given the urgency of need and the tremendous costs involved in developing new drugs, a good approach to therapeutic drug development is the repurposing of drugs known to accumulate in and affect the function of endolysosomes. The diprotic weak base drugs chloroquine (CQ) and hydroxychloroquine (HCQ), that de-acidify endolysosomes, have shown effectiveness in controlling SARS-CoV-2 infection in *in vitro* studies, however the effectiveness of CQ/HCQ against COVID-19 has not been established for COVID-19 patients (Liu et al., 2020; Wang et al., 2020; Yao et al., 2020). Endolysosome de-acidification can restrict replication of SARS-CoV-2 because acidic conditions are necessary for SARS-CoV-2 to enter into and be released from host cells. In the context of SARS-CoV-2 infection, CQ and HCQ have been used in combination with azithromycin (Andreani et al., 2020; Carlucci et al., 2020); a weak base antibiotic known to accumulate in endolysosomes (Kong et al., 2017; Choi et al., 2018b; Andreani et al., 2020). Of course, CQ and HCQ have other pharmacological actions, but the involvement of endolysosome de-acidification in SARS-CoV-2 infection is supported by findings that other endolysosome de-acidification drugs; ammonium chloride, bafilomycin A1 and monensin all block coronavirus infections at the entry-level (Hoffmann et al., 2020b; Pranesh et al., 2020; Yang and Shen, 2020).

However, de-acidification may have other unintended consequences that might result in increased viral levels. Acidic conditions in endolysosomes are necessary for TLR-induced type-I interferon-mediated antiviral immune responses and antigen presentation (Dalpke and Helm, 2012; Munz, 2012; Choi et al., 2018a; Viret et al., 2020). Acidic endolysosomes are also important for autophagy, which is important for initiating innate immune responses and the degradation of viruses (Dalpke and Helm, 2012; Choi et al., 2018a). Accordingly, de-acidification of endolysosomes might hamper autophagy-mediated antiviral responses (Kužnik et al., 2011) by deactivating RNA sensors (Belizaire and Unanue, 2009; Kužnik et al., 2011; Kazi et al., 2013; Hussman, 2020; Offerhaus et al., 2020; Schrezenmeier and Dörner, 2020). Therefore, improving innate immune responses using synthetic RNAs, oligonucleotides, or small agonists of TLRs as well as type-I interferon treatment might improve clinical responses to CQ and HCQ (Dalpke and Helm, 2012; Freund et al., 2019; Hussman, 2020; Lee and Shin, 2020).

## Inhibition of Coronaviruses at the Entry Level

The spike protein of SARS-CoV-2 is necessary for viral entry into cells governed by receptor-mediated endocytosis (Hofmann and Pöhlmann, 2004; De Clercq, 2006; Burkard et al., 2014; Burkard et al., 2014; Zheng et al., 2018; Jiang et al., 2020; Ou et al., 2020; Shang et al., 2020a; Tay et al., 2020). SARS-CoV-2 spike is a trimer with three receptor-binding domains (RBDs) of S1 heads on top of a trimeric S2 stalk (Gui et al., 2017; Shang et al., 2020a; Walls et al., 2020). Following proteolytic cleavage, the RBD of S1 conformationally switches from a laid-down position to a standing-up position in order to facilitate fusion with cell membranes (Hofmann and Pöhlmann, 2004; Gui et al., 2017; Yuan et al., 2017); the laid-down position has a significantly higher binding capacity (Walls et al., 2020; Wrapp et al., 2020) and escapes host immune surveillance. These features of the spike protein might make development of vaccines and antibody-based therapies more challenging (Figure 2) (Rossmann, 1989; Sui et al., 2014; VanBlargan et al., 2016; Gui et al., 2017; Chu et al., 2020; Xia et al., 2020). Never-the-less, huge efforts are on-going to develop vaccines and antibody-based therapies based on the structural and binding properties of RBDs (Jiang et al., 2005; Du et al., 2014; Shang et al., 2020b; Tai et al., 2020). Additional sites for intervention against viral infection include the spike S2 stalk that contains HR1 and HR2 hydrophobic regions; stable six-helix-bundle (6-HB) structures that fuse the virus with the host cell membrane (Figure 2) (Bosch et al., 2003; Bosch et al., 2004; Aydin et al., 2014; Wang et al., 2019). These mechanisms might represent sites for intervention against viral replication because targeting these hydrophobic regions has been shown to restrict infection of HIV-1 (Kong et al., 2016; Yuan et al., 2019), SARS-CoV-2, and other coronaviruses (Bosch et al., 2004; Xia et al., 2019a; Xia et al., 2019b; Wang et al., 2019; Xia et al., 2020).

Post-fusion with plasma membranes, many viruses enter cells by endocytosis and clathrin-mediated endocytosis (Inoue et al., 2007; Wang et al., 2008). Therefore it is not surprising that the anti-schizophrenia drug chlorpromazine (Ban, 2007) that inhibits clathrin-mediated endocytosis inhibits infection by the coronaviruses MHV (Pu and Zhang, 2008), MERS-CoV (Burkard et al., 2014), and SARS-CoV (Inoue et al., 2007; Wang et al., 2008). Similarly, Na^+^/K^+^-ATPase pump-based inhibitors bufalin and ouabain restricted MERS-CoV infection (Burkard et al., 2014; Burkard et al., 2015; Amarelle and Lecuona, 2018) by inhibiting clathrin-mediated endocytosis (Ko et al., 2020). An additional FDA approved drug that might find use against COVID-19 is camostat mesylate that is used for the treatment of pancreatitis (Ramsey et al., 2019); it inhibited serine proteases and restricted MERS-CoV, SARS-CoV, and SARS-CoV-2 infections by inhibiting TMPRSS2 activity (Figure 2) (Shirato et al., 2013; Bojkova et al., 2020; Hoffmann et al., 2020b). Also, the cathepsin L inhibitors Z-FY (t-Bu)-DMK, K11777, and teicoplanin blocked the entry of SARS-CoV and MERS-CoV (Huang et al., 2006; Adedeji et al., 2013; Zhou et al., 2016; Baron et al., 2020). Accordingly, the aboved named agents might find use against SARS-CoV-2 infection (Figure 2) and the pathogenesis of COVID-19.

## Effects of Endolysosome pH on Coronavirus Infection

The coronavirus spike protein is activated under acidic conditions by the endolysosome proteases TMPRSS2 and cathepsins B, L; conditions that promote fusion with host cell membranes and entrance into cells (Hoffmann et al., 2020b). Consistent with this, de-acidification of endolysosomes by CQ, bafilomycinA1, and ammonium chloride have all been shown to deactivate TMPRSS2 and cathepsin B, L as well as suppress coronavirus infection (Figure 2) (Simmons et al., 2004; Vincent et al., 2005; Wang et al., 2008; Shirato et al., 2013; Al-Bari, 2017; Gao et al., 2020; Hoffmann et al., 2020b). Although mentioned earlier, it is important to consider more specifically the involvement of endolysosome-resident ion channels and proteins that regulate endolysosome pH including TPCs, NPC1, and v-ATPase.

TPCs are calcium- and sodium-permeable channels that regulate cell membrane trafficking and endolysosome pH (Wang et al., 2012; Lagostena et al., 2017; Khan et al., 2020). Because of the involvement of TPCs in the regulation of endolysosome pH it is not surprising that TPC activation increased the entry and trafficking of SARS-CoV-2 (Ou et al., 2020), MERS-CoV (Gunaratne et al., 2018) and Ebola (Sakurai et al., 2015) while the TPC inhibitors tetrandrine and Ned-19 significantly inhibited the entry and trafficking of viruses in host cells (Figure 2) (Ou et al., 2020). Moreover, apilimod and vaculin-1 restricted SARS-CoV-2 infection by reducing PIKfyve enzyme activity (Kang et al., 2020; Ou et al., 2020); PIKfyve is a regulator of PI(3,5)P2, an endogenous activator of TPCs (Dove et al., 2009; Kirsch et al., 2018). Further, the natural flavonoid naringenin inhibited TPCs (Tsai and Tsai, 2012; Pafumi et al., 2017; Benkerrou et al., 2019; Bai et al., 2020) and has antiviral activity against hepatitis C (HCV) (Nahmias et al., 2008), influenza A (Dong et al., 2014), Zika (Cataneo et al., 2019), and Dengue (Frabasile et al., 2017). Additionally, naringenin suppressed acute inflammation by inducing lysosome-mediated degradation of inflammatory cytokines (Jin et al., 2017) and ameliorated radiation-induced lung fibrosis (Zeng et al., 2018; Zhang et al., 2018). Thus, naringenin and other drugs targeting TPCs might be considered as possible therapeutic strategies against COVID-19 (Figure 2).

Niemann-Pick disease type C1 (NPC1) is an endolysosome-resident protein (Higgins et al., 1999) that regulates trafficking of late endosomes and lysosomes (Ko et al., 2001; Zhang et al., 2001; Ganley and Pfeffer, 2006; Sztolsztener et al., 2012), membrane trafficking of essential cellular factors such as cholesterol and sphingolipids (Chen et al., 2005; Infante et al., 2008; Kwon et al., 2009; Lange et al., 2012; Höglinger et al., 2019), and regulation of endolysosome pH and calcium (Elrick et al., 2012; Liu and Lieberman, 2019; Wheeler et al., 2019a). Impaired NPC1 is an underlying cause of Niemann-Pick disease; a lysosome storage disease (Lloyd-Evans et al., 2008; Schuchman and Desnick, 2017). NPC1 has been implicated in the infectivity of Ebola virus, MERS-CoV and SARS-CoV following late entry kinetics and access to cathepsin L in late endosomes and lysosomes (Shah et al., 2010; Mingo et al., 2015; Zhou et al., 2016; Zheng et al., 2018; Ballout et al., 2020; ). Because SARS-CoV-2 also uses similar cell entry and cleavage mechanisms, NPC1 might become a target against SARS-CoV-2 infection; the desired effect being endolysosome de-acidification and accumulation of lipids in endolysosomes (Zheng et al., 2018; Wheeler et al., 2019a; Ballout et al., 2020; Pranesh et al., 2020; Sturley et al., 2020). Indeed, increased levels of 25-hydroxycholesterol (25-HC) restricted viral infection of *Filoviruses* (Liu et al., 2013), *Coronaviridae* (ZhangY. et al., 2019b), and *Flaviviridae* (Chen Y. et al., 2014; Li et al., 2017). Elevated levels of 25-HC and 7-ketocholesterol (7-KC) (Willard et al., 2018) in NPC compromised cells may restrict infection by SARS-CoV-2. Moreover, available NPC1 inhibitors U1866A and imipramine inhibited several enveloped viruses including MERS-CoV and SARS-CoV (Wrensch et al., 2014), Ebola (Herbert et al., 2015; Lu et al., 2015), HIV-1 (Tang et al., 2009), HCV (Elgner et al., 2016), influenza A (Eckert et al., 2014), and chikungunya (Wichit et al., 2017) by de-acidifying endolysosomes and increasing lipid accumulation (Lange et al., 2012) (Figure 2). Also, the anti-fungal drugs itraconazole and posaconazole are not only inhibitors of NPC but also have antiviral activity (Strating et al., 2015; Trinh et al., 2017; Meutiawati et al., 2018; Rhoden et al., 2018; Schloer et al., 2019; Takano et al., 2019a; Takano et al., 2019b). In addition, cepharanthine, an inhibitor of TPC2 and NPC1, has antiviral activity (Figure 2) (Zhang et al., 2005; Matsuda et al., 2014; Lyu et al., 2017; Bailly, 2019; Kim et al., 2019; Fan et al., 2020; Rogosnitzky et al., 2020). Thus, TPCs and NPC1 might both attract attention as possible targets to block SARS-CoV-2 infection and suppress COVID-19.

The v-ATPase pump is an ion channel that is crucial for regulating endolysosome pH (Mindell, 2012); higher or lower activity levels of v-ATPase significantly affects endolysosome functions (Colacurcio and Nixon, 2016; Halcrow et al., 2019a). CQ, Baf A1 and ammonium chloride all cause de-acidification and deactivation of proteases in endolysosomes as well as inhibit coronavirus infections (Vincent et al., 2005; Gao et al., 2020; Hoffmann et al., 2020b). The SARS-CoV 3CL^pro^ protease de-acidifies endolysosomes by direct interaction with the G1 subunit of v-ATPase (Lin et al., 2005) and blocks degradation of viral factors thereby enhancing virus replication. Endolysosome acidification may also restrict coronavirus infections by blocking the escape of viral RNA to the cytosol and promoting viral degradation in lysosomes (Carmona-Gutierrez et al., 2020; Gassen et al., 2020; Yang and Shen, 2020). A number of natural compounds acidify endolysosomes and might be tested for their ability to enhance coronavirus degradation; these include spermidine and spermine (Gassen et al., 2020), baicalein (Zhu et al., 2020), vitamin D3 (Hu et al., 2019; Daneshkhah et al., 2020), 17-beta-estradiol (Lipovka and Konhilas, 2014; Xiang et al., 2019; Khan, 2020; Suba, 2020), ketone bodies (Hui et al., 2012; Camberos-Luna et al., 2016), trehalose (Sharma et al., 2020), wogonin (Li et al., 2016), apigenin (Zhang X. et al., 2019), and butein (Ansari et al., 2018) (Figure 2).

## Conclusion

The high fatality rate of COVID-19 especially among people with pre-existing co-morbities and rapidly increasing case numbers of SARS-CoV-2 infections has created a huge global need for effective therapeutic interventions against COVID-19. Because of the urgent need for therapeutics, re-purposing already approved pharmaceuticals might be the quickest available strategy. SARS-CoV-2 enters into endolysosomes where it can escape detection by immune surveillance and from there can traffic to the cytosol where it can propagate. Endolysosomes generally and endolysosome pH more specifically may represent important targets against SARS-CoV-2 replication and COVID-19 pathogenicity, and several compounds and drugs are available that may be repurposed for immediate testing. Reviewed above were several potential targets to block SARS-CoV-2 infection including endocytosis following binding of the spike protein with its receptor (ACE2), RNA replication and transcription, translation and proteolytic processing of viral proteins, virion assembly, and release from infected cells (Guy et al., 2020; Poduri et al., 2020); all targets involving the endolysosome system. In considering approaches against SARS-CoV-2 infection and COVID-19 pathogenesis, the involvement of endolysosomes should be considered.

## Data Availability Statement

All datasets presented in this study are included in the article/supplementary material.

## Author Contributions

All authors contributed equally to the manuscript.

## Funding

P30GM100329, U54GM115458, R01MH100972, R01MH105329, R01MH119000, 2R01NS065957, and 2R01DA032444.

## Conflict of Interest

The authors declare that the research was conducted in the absence of any commercial or financial relationships that could be construed as a potential conflict of interest.
